# Using an Equine Cadaver Head to Investigate Associations Between Sub-Noseband Space, Noseband Tension, and Sub-Noseband Pressure at Three Locations

**DOI:** 10.3390/ani15142141

**Published:** 2025-07-19

**Authors:** Orla Doherty, Richard Conway, Paul McGreevy

**Affiliations:** 1 Life Sciences Department, University of Limerick, V94 T9PX Limerick, Ireland; orladoherty@live.ie; 2 Electronics and Computer Engineering Department, University of Limerick, V94 T9PX Limerick, Ireland; richard.conway@ul.ie; 3 Sydney School of Veterinary Science, University of Sydney, Sydney, NSW 2006, Australia

**Keywords:** equestrian, equitation science, social license to operate, noseband taper gauge

## Abstract

Pressures applied to horses via nosebands are of growing concern. The current study applied noseband pressure to the head of a dead horse. Pressure sensors were placed on the left nasal bone to record pressures as the noseband was progressively tightened. Tightness increased as predicated by holes in the strap of the noseband (as supplied) through eight steps from two fingers’ space, assessed using the standard International Society for Equitation Science Taper Gauge through to zero space. Sensors were also placed at the midline frontal plane and intra-orally at the level of the second premolar tooth. A strain gauge integrated into the noseband recorded tensions within the noseband at each tightness level, and a digital taper gauge under the noseband recorded forces on the face. Pressures at the left nasal bone rose to 403 kPa, while those at the frontal nasal plane reached 185 kPa. Pressures rose rapidly once the noseband was tightened at the equivalent of 1.4 fingers’ space under the noseband. These findings may help to explain cases of bone and skin damage at the noseband location and indicate the need to ensure that nosebands can accommodate more than the equivalent of 1.4 fingers beneath them in the nasal midline. Given that pressures are expected to rise from those reported here when horses wear bits, locomote, and when the reins are under tension, we conclude that the traditional provision of two fingers’ space should be retained.

## Simple Summary

The use of restrictive nosebands in equestrian sport is controversial. There are clear incentives for riders and coaches to use such nosebands to restrict mouth-opening in horses, even though these devices have been associated with compromised horse welfare outcomes. The International Society for Equitation Science (ISES) Noseband Taper Gauge is designed to demonstrate that the area under the noseband is sufficient to accommodate two adult fingers, between the nasal midline and the underside of the noseband, as recommended by traditional horse-riding texts. The current study reports on an investigation into changes in noseband tension and sub-noseband pressures at specific locations through the progressive tightening of a standard noseband on an equine cadaver. Using a probe that measured pressure, it assessed the area under the noseband using the ISES Noseband Taper Gauge. Pressures rose rapidly once the noseband was tightened with the equivalent of 1.4 fingers’ space or less under the noseband. These findings may help to explain why nosebands should accommodate more than the equivalent of 1.4 fingers beneath them in the nasal midline. Given that pressures are expected to rise from those reported here when horses wear bits, locomote, and when the reins are under tension, we conclude that the traditional provision of two fingers’ space should be retained.

## 1. Introduction

Concern for possible threats to horse welfare due to tightened nosebands has been expressed over several years [1,2,3,4,5]. Riders may be motivated to tighten nosebands because rein tension data suggest that doing so increases responsiveness to the aversive pressures from the bit [6]. Most horse training techniques use rider-applied pressure to evoke a specific response (negative reinforcement), but effective training requires the release of such pressure once the horse gives the correct response [7]. In contrast, pressures exerted by a tight noseband persist throughout ridden work and cannot be removed by performance of a correct or desired response.

The use of tight nosebands has been prevalent in the disciplines of dressage and eventing [3], although growing awareness of this issue may be resulting in changing trends, with fewer tight nosebands found in more recent studies [8,9]. Recent exposés of changes in the colour of tongues in elite dressage horses [10,11] may have added to interest in the interactions between gear and ridden horse welfare.

While anecdotal evidence suggests that occasional periosteal damage and bony deformation at the location of noseband position [12] and laceration of the mucosa where the noseband passes over the second premolar tooth possibly pressing sensitive soft tissue against sharp tooth edges occur [13], insufficient evidence exists to identify the exact mechanism and aetiological factors that contribute to this outcome.

Tight nosebands have been shown to lead to a possible reduction in vascular perfusion of skin in the region of the noseband [5]. Radiographic studies of cavalry horses (n = 144) have revealed the presence of bony changes at the level of the noseband in approximately 33% of horses, with evidence of bone thinning and bone deposition on the nasal bones and mandibular rami at the normal location of the noseband [14]. Pressure exerted on tissue can result in tissue deformation and tissue damage [15,16]. Hair loss under the noseband has been reported as the most common noseband-related complication noticed by owners, riders, and trainers [17].

While it has been proposed that more behavioural and physiological studies with nosebands tightened at different levels would have merit [18], several such studies have been published showing physiological stress responses to restrictive nosebands including increased heart rate, reduced heart rate variability [2], and an increase in eye temperature, as measured by infrared thermography [2,19]. In Fenner et al.’s study [2], tight nosebands restricted oral behaviours (licking, yawning, chewing, and swallowing) that showed a post-inhibitory rebound once the noseband was loosened. This implies that such behaviours are internally motivated and that denying them is distressing. In their study of 3143 horses, Uldahl et al. found that a looser noseband was associated with fewer lesions at the lip commissures, with increasing space under the noseband from <2 to 2–3 cm or from 2–3 to >3 cm, lowering incidence of lesions by 34% (odds ratio, 0.66; 95% confidence interval, 0.51–0.86; *p* = 0.002) [20]. It is important to note that the populations of horses studied and the methods applied vary across the different studies [2,18,19].

Tight nosebands have been used to induce a stress response (as inferred by increased cortisol concentrations) experimentally, during which an increase in stress-related behavioural responses was also recorded [21]. That said, one study of horses (n = 8) that had their nosebands tightened for 20 s before being offered a treat reported no effect of noseband tightening on eye temperature or blink rate [22]. It is unclear why such a brief period of study was selected or how the horses’ motivation to accept a treat and chew while jaw movement was restricted by noseband tightening was standardised or, for that matter, manipulated.

Analysis of the anatomy of the head of the horse, at the usual position of a cavesson noseband, revealed the left and right nasal bones and the mandibular rami to be the locations most susceptible to high levels of pressure from a tightened noseband [1]. In practice, the working guideline based on tradition, rather than empirical data, is that one must be able to fit two fingers between the noseband and the frontal nasal plane [23,24]. The mean height of the midpoint of the intermediate phalanx of the digitus medius is 1.59 ± 0.05 cm [10]. The mean width of the midpoint of the intermediate phalanx of the digitus secundus and digitus medius side by side is 3.87 ± 0.09 cm [10]. These details are provided to explain why the current article refers to the area under the noseband in terms of fingers (noting that the cross-sectional area of two adult fingers was 3.87 cm × 1.59 cm = 6.1533 cm^2^ [10]).

Tight nosebands have been shown to impose high forces (of up to 95 N) and peak pressures of more than 1000 mm Hg (133.32 kPa) on supporting tissues [1,25,26]. While varying technology, methodology, and statistical analyses are used in different studies, the common finding of such high pressures on sub-noseband tissues warrants further detailed empirical investigation.

A stated goal of the FEI Code of Conduct for the welfare of the horse states that “At any level of competition, a noseband may never be so tightly fixed that it causes harm to the horse”. FEI tack stewards were historically advised to check noseband tightness by introducing an index finger between the horse’s cheek and the noseband. However, the contour of the face at the cheek is such that noseband pressures at this location are only approximately one-third of those measured at the frontal nasal plane, even for the tightest noseband fitting [26]. This allows for the easy introduction of a finger/fingers at that location, rendering that measurement unrepresentative of the actual pressures exerted on sub-noseband tissues at other critical areas on the head, particularly at the left and right nasal bones and mandibular rami.

As a result of ongoing widespread concerns about tight nosebands and the need to regulate noseband tightness, equestrian federations in Denmark, Sweden, New Zealand, Switzerland, and the Netherlands have introduced regulations regarding noseband tightness. While all the above countries specify that the tightness level should be measured at the frontal nasal bone, there are differences in the methods of measurement used. To date, the taper gauge approach has not been checked with the use of a digital tightness gauge beneath the noseband. Recently, the FEI has announced a requirement to measure noseband tightness on the dorsum of the nose and the introduction of a “pull-through” gauge that appears to equate to a maximum tightness of approximately 1.5 fingers [27]. This requirement came into force in 2025.

The magnitude of force directed by a noseband downward onto underlying tissue will depend on the local morphology and compliance of structures underlying the noseband and the degree of tightness with which the noseband is fastened, as shown by the Law of Laplace below [28].

Anatomical areas most susceptible to forces exerted by overly tight nosebands have been identified using curvature models of an equine nose and verified experimentally [1,25,26]. Areas with a high degree of curvature are subject to higher compressive forces than areas of lower curvature. Locations of high curvature include the left and right nasal bones, the left and right mandibular rami, and the lateral vestibular or cheek region covering the premolar teeth. The hammocking effect concentrates the noseband force onto the “peaks” of the bony prominences. These areas are particularly subject to noseband-induced normal forces due to their rigidity. Similarly, the application of concentrated forces onto relatively small areas, such as the “peaks” of the bony prominences, implies that the force per unit area, i.e., the pressure at these locations, will also be elevated. While mapping of the distribution of nociceptors around the face of the horse has yet to be performed, data regarding the high prevalence of nociceptors in the periosteum exist [29], suggesting that the periosteum of the nasal bones and mandibular rami are susceptible to sustained pressure levels, which may elicit pain. While the hammocking effect concentrates the noseband force onto the peaks of the bony prominences, with tissue damage most likely to occur at these locations, sustained pressure is also exerted, albeit with lower levels of force, against tissues and structures of lower convexity (smaller radius of curvature) [1].

We sought to explore the relationships between pressure on the nasal bones and “number of fingers” on the ISES Taper Gauge and between pressure on the nasal bones and noseband tension. Despite the limitations of the current pilot study, the latter objective, if validated, would allow future researchers to infer pressure from tension data. This is helpful because noseband tension (or tightness level) is easier for horse owners/riders to measure (such as by inserting fingers under the noseband or using a tightness measuring gauge) than sub-noseband pressure (which requires access to pressure-measuring technology). The current study may be considered one approach to understanding how pressure beneath the noseband can be inferred from tension data. Eventually, if the use of restrictive nosebands persists, further research may help clarify the roles of the contact area of the noseband, the compliance of the noseband’s core, and the area, thickness, and physical properties of the padding when estimating the pain or discomfort these devices can impose on horses.

The goals of the current cadaver study were 1. to measure the levels of pressure exerted by an incrementally tightened noseband on the nasal bone, at the frontal nasal plane and at the location of the second premolar tooth; 2. to measure objectively noseband tension, as the noseband was progressively tightened from loose to the tightest possible fitting; and 3. to investigate the impact on noseband tension of inserting a digital tightness-measuring gauge beneath the noseband.

The use of a cadaver was chosen because of the ethical unacceptability of tightening the noseband to zero fingers’ space on a sentient living animal. A cadaver was also chosen because it represents the passive situation, i.e., in the absence of living soft tissues under systolic pressures. It is possible that pressures and tensions would be higher in a live horse and higher still when the horse is ridden in a contact.

The rationale that underpins this expectation is based on the knowledge that there is no space in the buccal cavity that has evolved to accommodate a bit or bits. As such, the bit represents a foreign body. The tongue is a muscle, and, in a live horse, it is active in the presence of a bit. The bitted horse attempts to seek comfort by morphing the tongue’s shape and, where possible, opening the mouth and, when denied this behaviour, by simply attempting to open the mouth. These outcomes were noted by Clayton and Manfredi, whose fluoroscopic study revealed that, when bitted, horses spent “less time quiet [than when not bitted] and more time mouthing the bit, retracting the tongue and bulging the tongue over the bit when tension was applied” [30]. Noseband adjustment in a dead horse can act as a baseline before the addition of rein tension in live horses. This is logical since reins are designed to make bit pressure relatively more aversive to incentivise the horse to respond to the rider. Once bit pressure is increased, so is the horse’s need for comfort behaviours. A live, active tongue cannot decrease noseband pressures and tensions, nor can rein tension. Based on this logic, our expectation is that these metrics would not fall but would instead rise when live horses are exposed to any rein contact.

## 2. Materials and Methods

### 2.1. Apparatus

The head and rostral third of the neck (to approximately C3) of an adult crossbred horse were acquired immediately following death by traffic accident (that had not damaged the structures of interest for the current study). The specimen was stored in a freezer from the day of collection until 24 h prior to data collection. Following removal from the chill, hydrogen peroxide was used to remove blood from the external surfaces of the head. The exposed muscle and spinal region were sealed using Elastoplast^TM^ bandage (Beiersdorf, Hamburg, Germany), plastic membrane, and adhesive tape. A stainless-steel skewer (50 cm long, 1 cm diameter) was introduced through the cervical skin and muscle mass, ventral to the vertebral column.

**Figure 1:**
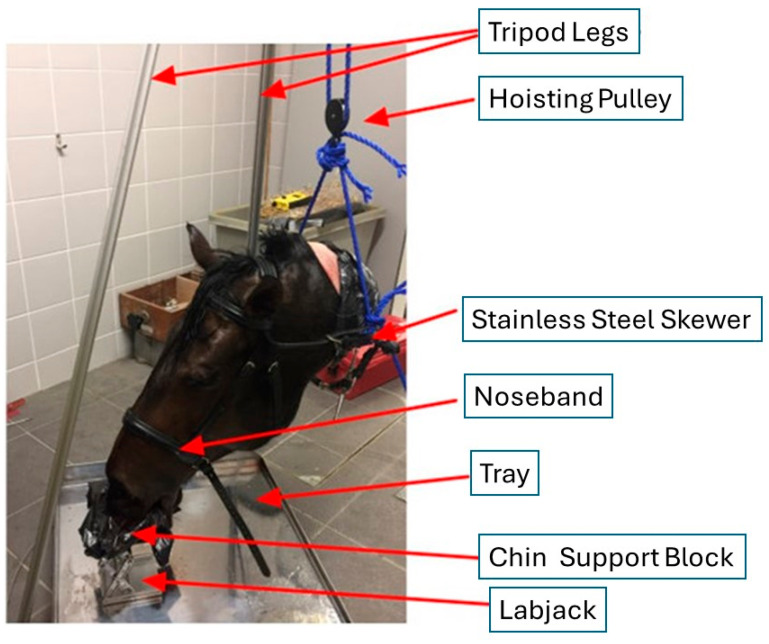
An aluminium tripod from which the cadaver head was suspended, allowing noseband and sensor adjustments to be conducted. The chin support block limited rotational movements of the head during data collection.

A tripod was assembled using aluminium profile bars (20 mm × 20 mm), drilled and joined by wire at the apex and stabilised by connecting fixed lengths of wire between adjacent legs at floor level (Figure 1). A laboratory pulley wheel (6 cm diameter) with a steel hook was used to support the head using nylon rope (0.8 cm diameter) secured to the extending ends of the stainless-steel skewer. This arrangement allowed for easy adjustment of the head position within the tripod to resemble normal equine head carriage (with the frontal nasal plane slightly in front of the vertical). The rostral extremity of the head was supported by the placement of a padded block on a standard adjustable laboratory jack.

**Figure 2:**
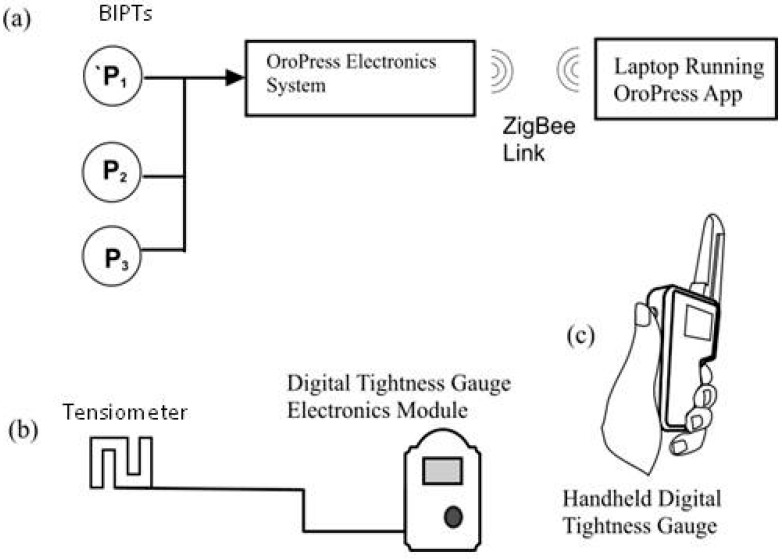
Schematic representation of the electronic instrumentation systems used in the study: (**a**) Oropress biomedical interface pressure transducers (BIPTs), (**b**) S-type loadcell tensiometer based on a digital taper gauge electronics module, (**c**) and a Digital Tightness Gauge (DTG).

**Figure 3:**
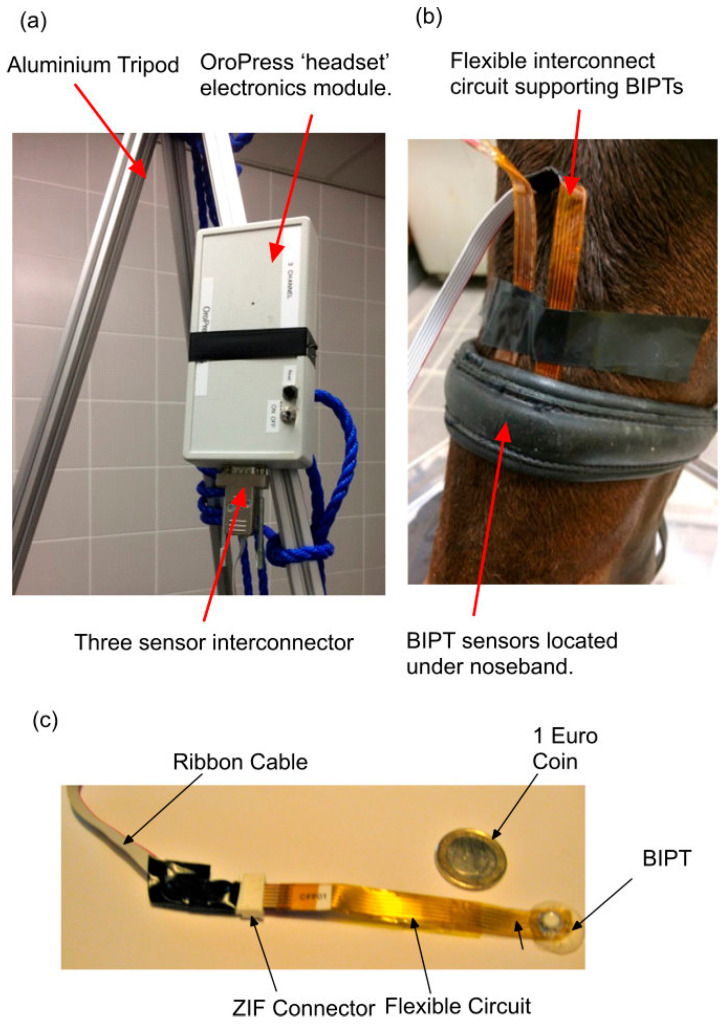
(**a**) Oropress wireless pressure measurement system, (**b**) BIPT placement under noseband left nasal bone and medial position at frontal nasal plane, and (**c**) BIPT probe showing flexible circuit and ZIF (Zero Insertion Force) connector linkage to standard ribbon cable.

Three independent electronic instrumentation systems were used: sub-noseband pressures, noseband tension, and noseband tightness (Figure 2). Pressure at noseband–tissue and tissue–tissue interfaces was monitored using contoured biomedical interface pressure transducers (BIPTs), measuring 16 mm in diameter and 3.5 mm high at centre sensor location [31]. These were supported by flexible printed circuit substrates (Figure 3c) and connected to an Oropress unit [32], which connected wirelessly with a laptop running the Oropress application. The Oropress app displayed and logged the pressure and temperature for up to three biomedical interface pressure transducer (BIPT) sensors simultaneously at a rate of 30 readings per second (Figure 3).

**Figure 4:**
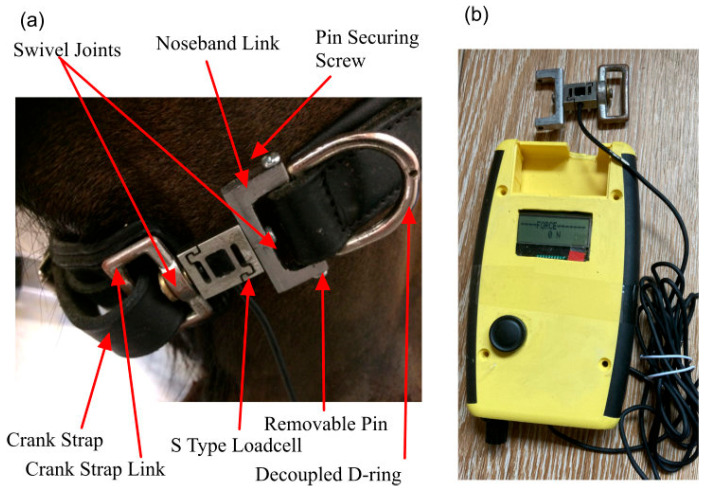
(**a**) S-type loadcell strain gauge connected to cavesson noseband straps with swivel joint. (**b**) Modified digital taper gauge connected to S-type loadcell strain gauge.

A standard cavesson bridle (Elevator Bridle, XBSELS-F16BK, Full Size, E. Jeffries & Sons Ltd., Walsall, UK), from which the bit was removed, was fitted onto the head. The noseband used was a raised crank noseband, consisting of two leather straps, a dorsal strap, and a ventral strap normally linked using “D-rings”. The dorsal strap had padding (approx. 3 mm depth) along the entire length, while the ventral strap also had a widened padding addition (approx. 45 mm by 120 mm, approx. 5 mm depth) spanning the mandibular rami. The D-ring on the right latero-ventral aspect, through which the tightening strap of the noseband passes, was replaced with an “S-type” loadcell or strain gauge with swivel-jointed links to allow for mechanical integration with the noseband (Figure 4). The strain gauge was connected to a modified DTG circuit [27], which allowed for calibration over a 0–300 N force range and mode switching to display the load. These were linked via a Bluetooth wireless link to an Android app for calibration, as desired. Correct force magnitudes were obtained through calibration of the measuring device with reference weights, using known masses to generate a standard force. Repeatability was achieved using multiple force measurements under the same conditions. A standard deviation of less than 0.5% was obtained using three repeated sets of measurements.

The noseband had been manufactured with 14 standard holes pre-punched at approximately 1.5 cm distance from each other to allow tightening. A record of the tightness level (using hole number) was maintained during each test. Each sensor was placed directly beneath the path of the cavesson noseband, and the externally placed sensors were held in place using adhesive tape. Adhesive tape was also used to secure the circuitry caudal to the sensor (Figure 4).

**Figure 5:**
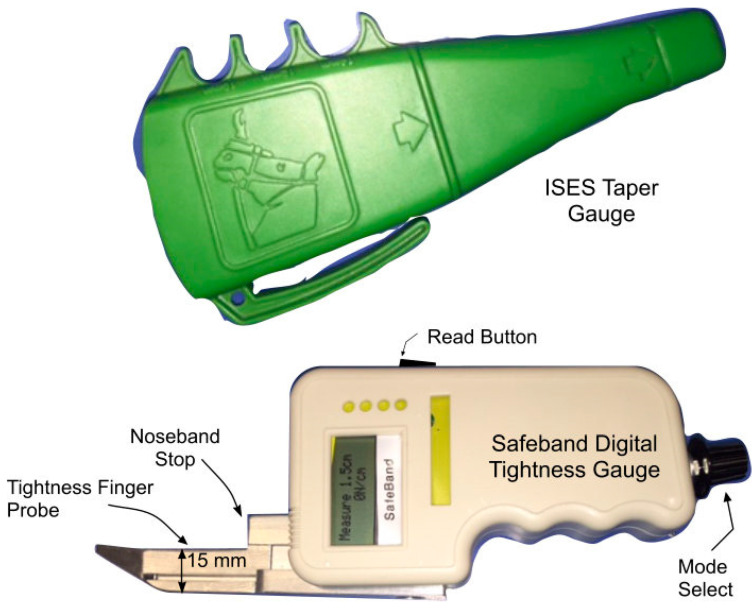
ISES Noseband Taper Gauge (**upper image**) and SafeBand Digital Tightness Gauge (**lower image**) designed to provide an objective measure of noseband tightness, displayed digitally after the probe has been placed beneath the noseband (Doherty, 2017) [26].

The ISES Noseband Taper Gauge (Figure 5) was used to estimate the tightness of the noseband at each stage of tightening. This device was designed to allow riders, coaches, and tack inspectors at competitions to measure and regulate noseband tightness levels and tapers smoothly from one finger to two finger diameters, based on a sample of ten adult males and ten adult females, with a circumference of 102 mm [5].

Prior to conducting Tests 1–3, the noseband was tightened through Holes 6 to 14 and, at each hole, the approximate space under the noseband was measured, as indicated by the insertion of the ISES Noseband Taper Gauge, used in the recommended fashion, at the frontal nasal midline (see Table 1). The approximate space under the noseband was based on an estimate, as the ISES Taper Gauge was designed to gauge two and one fingers’ space, and it was not calibrated for other space measurements, although its use for such estimates has been published in the peer-reviewed literature [3,18]. Zero fingers’ tightness was used to describe the noseband tightness settings at which the tip of the ISES Taper Gauge could not be introduced under the noseband at the midline frontal nasal plane.

During each test, the noseband was tightened progressively with the buckle keeper placed in consecutive pre-punched holes along the noseband. The holes were numbered from 1 (the first hole on the loosest edge of the noseband) to 14 (the tightest). It was observed that no measurable pressure was exerted by the noseband until Hole 6 was engaged to fasten the buckle. Therefore, in this and all subsequent tests, measurement began at this hole, which was described as “loose”. The tightness of the noseband increased when the holes beyond this number were used. In Test 1, the tightest fitting achieved was Hole 13. During Test 2, the noseband was tightened to Hole 14.

#### 2.1.1. Test 1: Sub-Noseband Pressures as a Function of Noseband Tightness

To identify noseband-imposed pressures at three locations, pressure sensors were placed as follows:

- At the left nasal bone;
- On the second premolar tooth, with the sensor facing the buccal mucosa;
- At the midline frontal nasal plane.

The noseband was progressively tightened from Holes 6 to 13. At each tightness level, data pressure (kPa) was recorded from each of the three pressure sensors.

#### 2.1.2. Test 2: Sub-Noseband Pressures and Noseband Tension as a Function of Noseband Tightness

Test 2 repeated the measurements taken in Test 1, but with the internally placed sensor located at the second premolar tooth facing the tooth surface, while noseband tension was also measured as the noseband was progressively tightened.

Pressure sensors were placed in three positions as follows:

- At the left nasal bone;
- On the second premolar tooth, facing the tooth surface;
- At the midline frontal nasal plane.

The noseband was progressively tightened from Holes 6 to 14. At each tightness level, two sets of data were recorded as follows:

1. Tension (Newton), as recorded by the strain gauge;
2. Pressure (kPa) from each of the three pressure sensors.

#### 2.1.3. Test 3: Investigating the Effect of Placing a Digital Tightness Gauge Beneath the Noseband on Recorded Noseband Tension Levels

**Figure 6:**
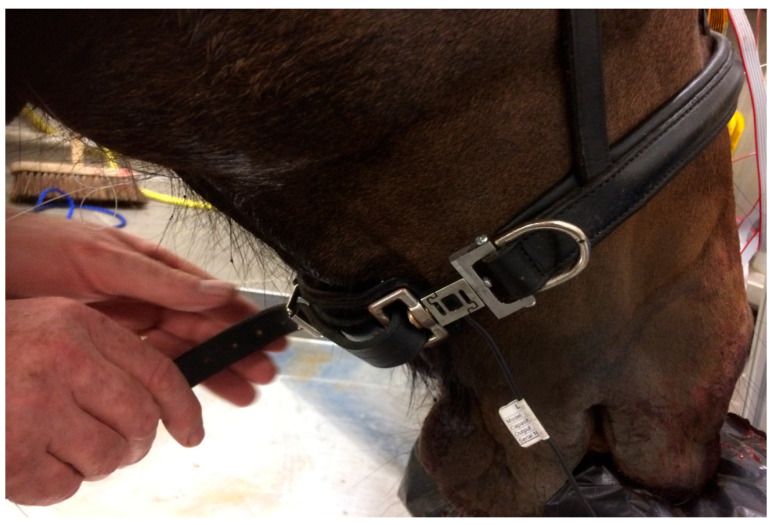
During each test, the noseband was tightened progressively with the buckle keeper placed in each of the consecutive pre-punched holes along the noseband, with the inbuilt noseband strain gauge measuring increases in noseband tension at each tightness level.

Using the digital tightness gauge, tightness levels were measured as the noseband was progressively tightened from Holes 7 to 12, the tightest noseband fitting which still allowed sufficient space under the noseband to allow the DTG to fit underneath the noseband to collect data At each setting, noseband tension was recorded from the noseband tensiometer/strain gauge, both prior to insertion of the digital taper gauge and after insertion of the digital taper gauge. At each data collection point, once data were collected, the digital taper gauge was removed, and the noseband was tightened to the next hole (see Figure 6). It was not possible to fasten the noseband tighter than the 1.0 finger setting (Hole 13 or 14) for this test because, to sense the load, the DTG lifts the noseband away from the nose, and a tighter noseband would not accommodate the DTG beneath it. Measurements at less than Hole 7 were not possible because the noseband was too loose.

### 2.2. Physics Considerations

**Figure 7:**
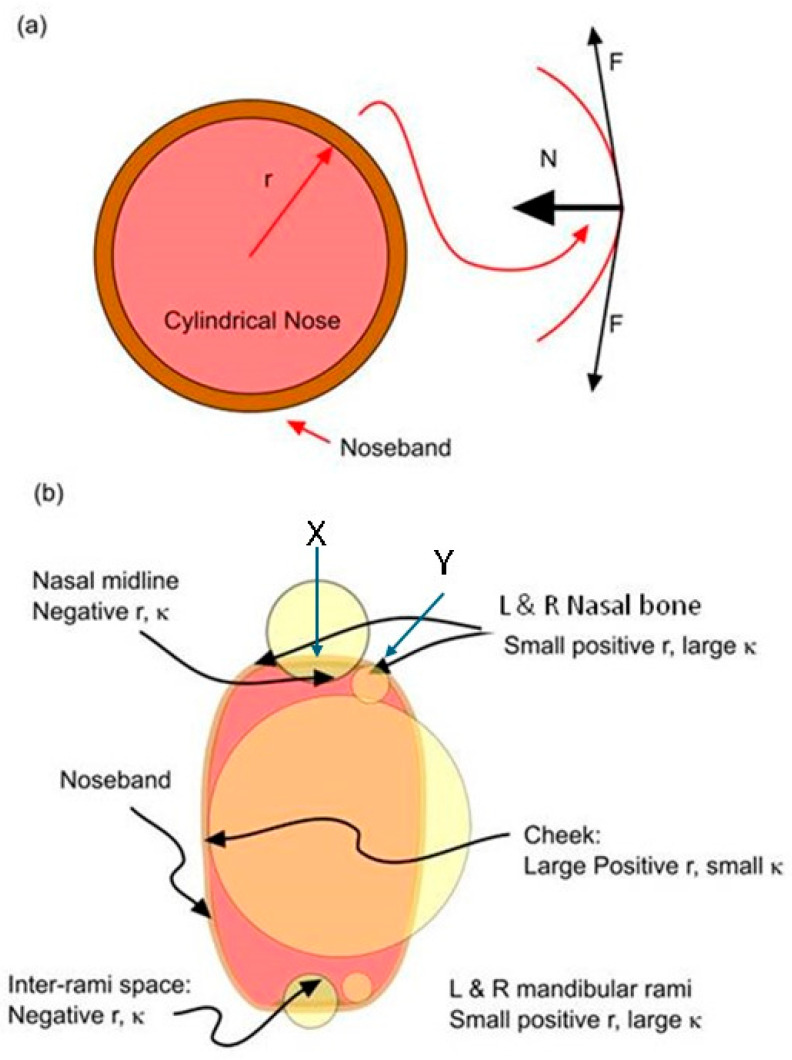
(**a**) Idealised cylindrical nose with snugly fitted noseband illustrating normal force produced by tension in encircling noseband. (**b**) More realistic diagrammatic cross-section of the equine nose showing regions of positive (load bearing) and negative (hammocking) curvature. The yellow circles indicate areas of differing radius of curvature. Areas of hammocking occur where there is a negative radius of curvature [X]. Areas of load bearing occur where there is a positive radius of curvature [Y]. r = radius of curvature, k = 1/r.

To illustrate the effect of curvature on sub-noseband forces and pressures, it is convenient to consider a highly idealised equine nose geometry—that of a regular cylinder (Figure 7).

A loose noseband has a circumference/perimeter larger than the circumference of the nose beneath it. This will result in zero tensional force, provided the mouth remains closed. Tightening a noseband reduces its effective circumference, bringing it closer in value to that of the nose. In cases of very tight nosebands that cause tissue deformation, the noseband circumference may be smaller than the unrestricted nose circumference. In circumstances where the nose circumference is greater than that of the noseband, either through an over-tightened noseband or through normal mouth actions, e.g., tongue movement, chewing, and yawning, a tensional force will be produced in the noseband.

If we consider the nose as having a simple cross-sectional geometry corresponding to a disc, i.e., constant radius of curvature, the tensional force will produce a centrally directed/normal component of force (N) directed against the underlying tissue, which will depend directly on the magnitude of the tensional force (*T*) and inversely on the radius of curvature, *r* (assuming a positive or convex structure), of the local support structure according to the law of Laplace [28], which may be expressed as follows:

$$P\propto \frac{T}{r}\;\equiv T=MTĸ$$

where $ĸ=\frac{1}{r}$. A multiplier constant *M* is generally added to provide an equation which gives the average sub-band pressure in convenient units, e.g., kPa.

Tightening of the noseband can result in compression of the sub-noseband tissue, where tissues are soft and can deform in response to the applied force. Where the profile of the face contains a hollow or concave area (negative curvature), such as between the mandibular rami, nasal bones, and dorsal to the vestibular arcade, the noseband will stretch unsupported over these areas but will relay the accumulated force per unit length onto the elevated structures on either side of the lift-off zone in much the same way that a hammock’s load is concentrated at its attachment points. This is commonly referred to as the hammocking effect [33,34].

Approximate space under the noseband at the frontal nasal plane (using the ISES Noseband Taper Gauge) at 9 noseband fittings is shown in Table 1. As the ISES Noseband Taper Gauge is only calibrated to measure space sufficient to allow 1 and 2 fingers fit in that space, the results shown are estimates made by observing how far the taper gauge could be easily inserted, using approximately the same amount of force and carried out by the same person.

## 3. Results

### 3.1. Test 1: Sub-Noseband Pressures as a Function of Noseband Tightness

This section of the study involved measuring pressure levels at three different anatomical locations as the noseband was tightened (see Table 2).

Pressure on the buccal mucosa at the location of the second premolar tooth increased from 0.07 kPa at noseband Hole 6 (loose) to 7.13 kPa at the tightest noseband fitting (Hole 13). The corresponding hole positions produced an increase in pressure from 0 to 185.2 kPa at the frontal nasal plane midline and from 0 to 403.19 kPa at the left nasal bone location. The gradients of the linear fits to the data provide rates of the change in tension per finger change on the ISES Gauge.

**Figure 8:**
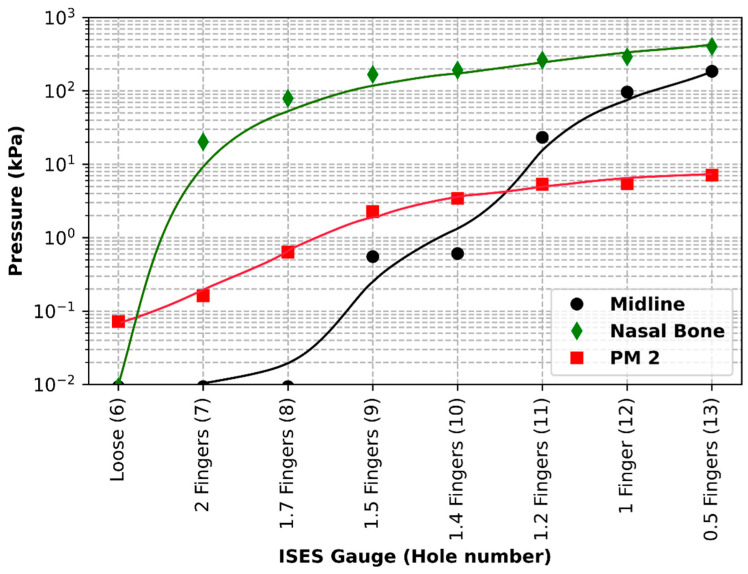
Sub-noseband pressure at three locations as a function of noseband hole setting, as the noseband is tightened from hole No. 6 to hole No. 13. Pressures at the left nasal bone (green line) rose to 403 kPa at zero fingers’ tightness (Hole 13), with pressure at the midline frontal nasal plane (black line) and at the second premolar tooth (PM 2—red line) rising to 185 kPa and 7.1 kPa, respectively, at zero fingers’ tightness.

Pressure data (kPa) from the three locations are shown on a semi-log plot (as pressures ranged from 0 to in excess of 400 kPa, i.e., two orders of magnitude (see Figure 8)). The persistence of low-pressure levels at the midline as the noseband was tightened through Holes 6 to 8 represents the hammocking effect. Due to their prominence, noseband pressure is exerted mainly on the nasal bones on either side of the midline.

**Figure 9:**
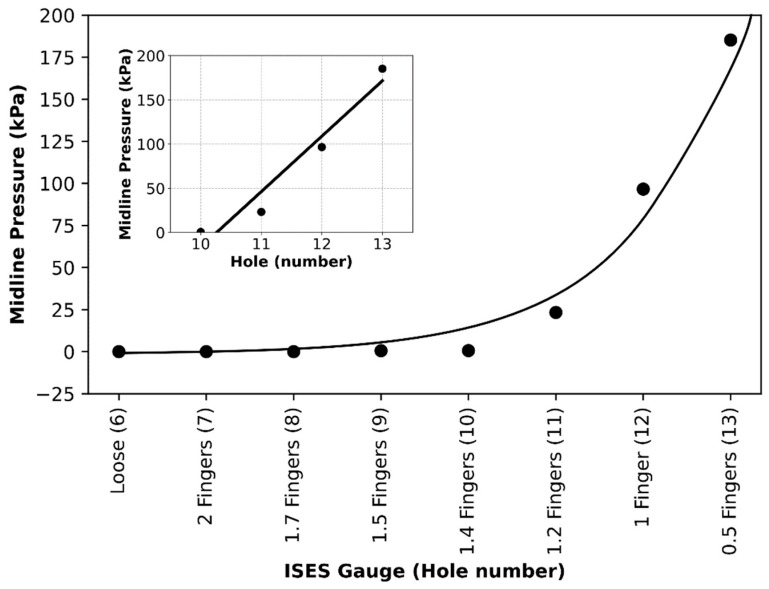
Midline data fitted to an exponential growth model. The insert shows a linear fit to the data subset outside of the hammocking region. The insert shows the “tight” noseband pressure data (Holes 9 to 13) at the midline location, which gives a reasonable linear fit (P = 62.66 H − 643.89 kPa, R^2^ = 0.92), indicating a pressure rise of 62.66 kPa per hole in this region.

Regression analysis of the full midline data set revealed an excellent fit to an exponential growth model; $P=0.002e^{0.871\times H}$ and R^2^T = 0.910 (Figure 9). However, the exponential growth model may be an artefact of the hammocking effect.

**Figure 10:**
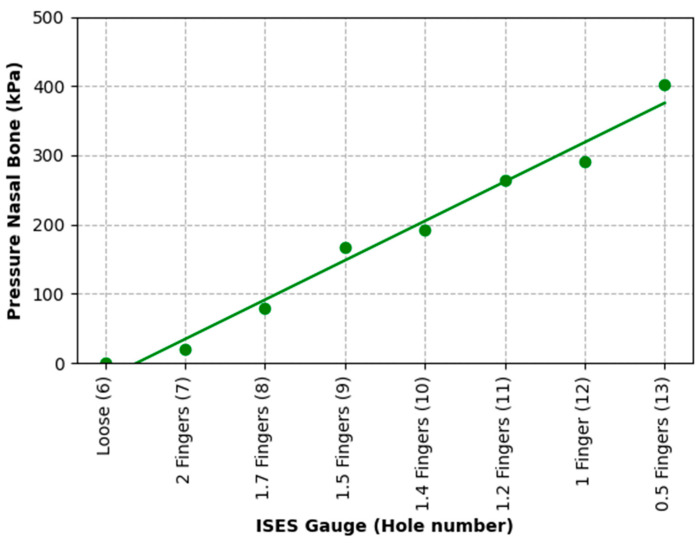
Linear model fitted to nasal bone pressure data as a function of noseband tightness as judged by hole number. Pressure increased by an average of 56.93 kPa per hole as the noseband was tightened.

The nasal bone pressure data are plotted in Figure 10 and fit a linear model (P = 56.93 H − 360.3, R^2^ = 0.96) with a pressure rise of 56.93 kPa/hole.

**Figure 11:**
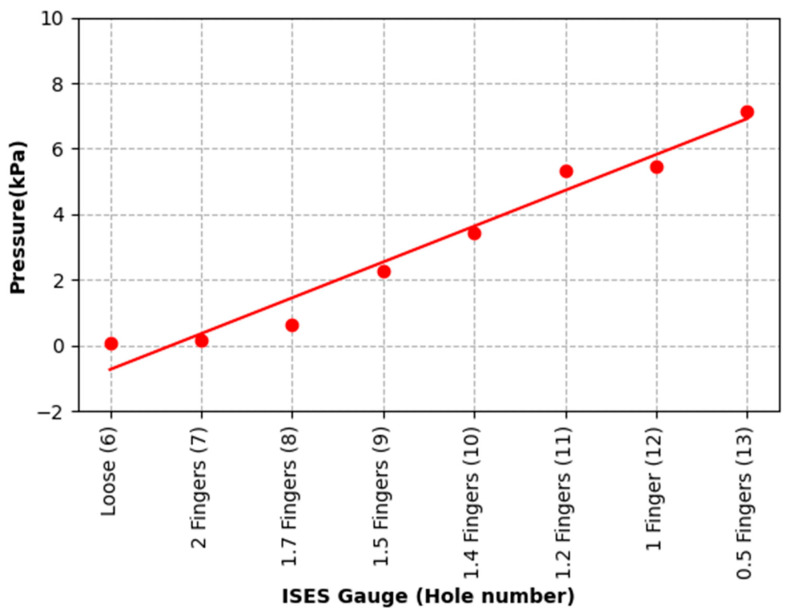
Pressure data detected at Holes 6 to 13 from the internal sensor located between premolar 2 and the buccal mucosa. Pressure rose from zero at Hole 6 to 1.09 kPa at Hole 13.

The pressure data for the second premolar tooth sensor location (see Table 3) are plotted in Figure 11 and fit to a linear model (P = 1.09 H − 7.29) kPa throughout the range of noseband tightness tested; R^2^ = 0.95.

### 3.2. Test 2: Sub-Noseband Pressures and Noseband Tension as a Function of Noseband Tightness

In this test, pressures beneath the noseband at three locations (frontal nasal plane, left nasal bone, and at the second premolar tooth, with the sensor facing the tooth surface) were measured alongside the noseband tension (N) at nine different noseband tightness levels, from loose to zero fingers (see Table 3).

**Figure 12:**
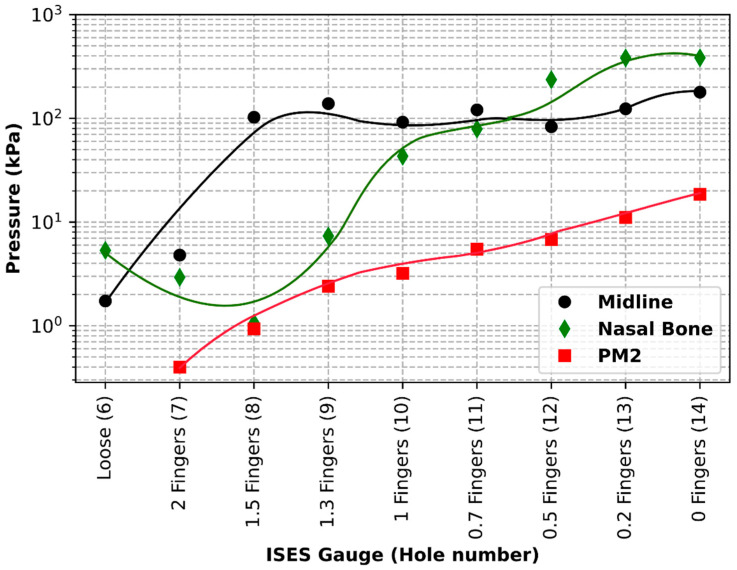
Sub-noseband pressure at three locations as a function of noseband hole setting, as the noseband is tightened from hole No. 6 to hole No. 14. Pressures at the left nasal bone (black line) rose to 383 kPa at zero fingers’ tightness (Hole 14), with pressure at the midline frontal nasal plane (green line) and at the second premolar tooth (PM 2—red line) rising to 178.91 and 18.53 kPa, respectively, at zero fingers’ tightness.

A plot of the pressures (kPa) recorded at the second premolar tooth, midline at the frontal nasal plane, and at the left nasal bone can be seen in Figure 12.

**Figure 13:**
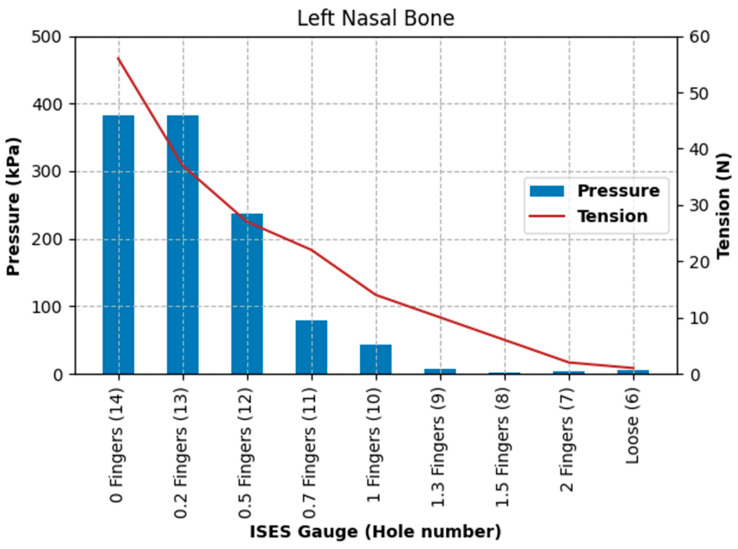
Pressures (kPa) recorded by the pressure sensor under the noseband at the left nasal bone and noseband tension (N) measured by the inbuilt noseband strain gauge as the noseband was progressively tightened from loose to zero fingers’ tightness, as indicated by the ISES Taper Gauge.

Pressure at the left nasal bone increased from 5.3 kPa at the loosest fitting, with a noseband tension of 1 N, to 383.3 kPa at the tightest fitting (zero fingers), with a noseband tension of 56 N, as shown in Figure 13.

**Figure 14:**
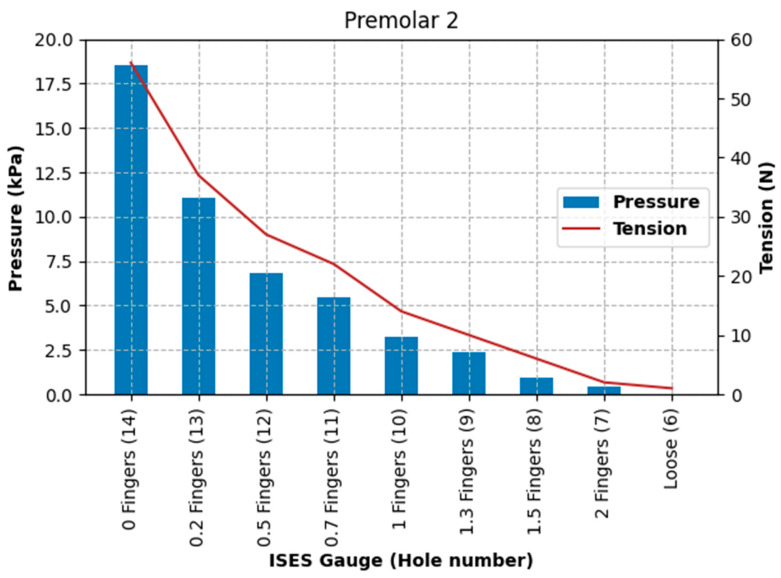
Pressures (kPa) recorded under the noseband at the location of the second premolar tooth and noseband tension (N) as the noseband is progressively tightened from loose to zero fingers’ tightness as indicated by the ISES Noseband Taper Gauge.

Pressure at the position of the second premolar tooth increased from 0 kPa at the loosest fitting (2.0 fingers), with a noseband tension of 1 N, to 18.53 kPa at the tightest fitting (zero fingers), at a noseband tension of 56 N (see Figure 14).

**Figure 15:**
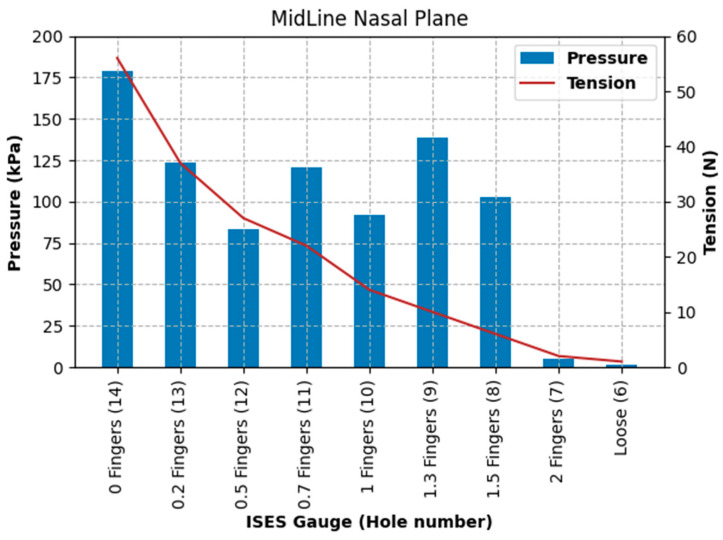
Pressures (kPa) recorded under the noseband at the frontal nasal plane (midline) and noseband tension (N) as the noseband is progressively tightened from loose to zero fingers’ tightness, as indicated by the ISES Noseband Taper Gauge.

Pressure at the frontal nasal plane increased from 1.73 kPa at the loosest fitting (two fingers), with a noseband tension of 1 N, to 178.92 kPa at the tightest fitting (zero fingers), with a noseband tension of 56 N (see Figure 15).

### 3.3. Test 3: Investigating the Effect of Placing a Digital Tightness Gauge Beneath the Noseband on Recorded Noseband Tension Levels

**Figure 16:**
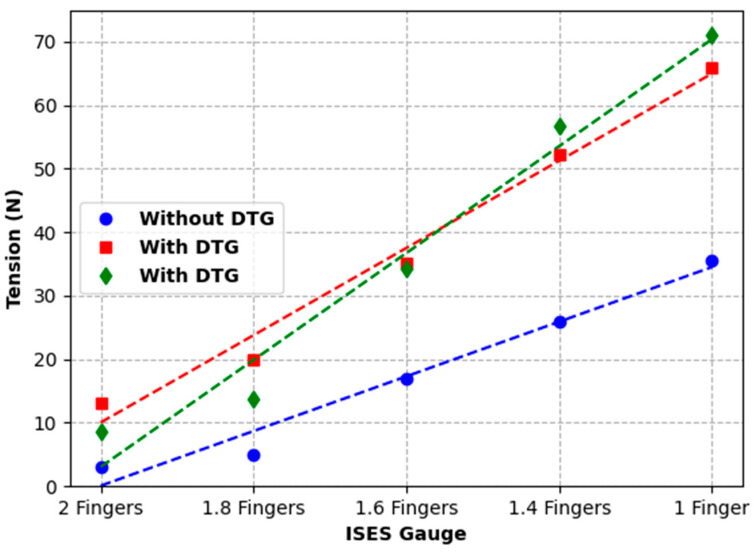
Measuring the effect of the introduction of a measuring tightness gauge (the DTG) underneath the noseband at 5 noseband tightness settings. The lowest (blue line) shows the noseband tension measured by the noseband strain gauge at 5 ISES Noseband Taper Gauge tightness settings before the introduction of the digital taper gauge (DTG) beneath the noseband. The red line shows the noseband tension levels measured by the strain gauge at each noseband tightness setting, after the introduction of the DTG beneath the noseband. The green line shows the noseband tension measured by the DTG underneath the noseband at the same 5 settings.

Plots of the noseband tension, as measured by the inbuilt noseband strain gauge, versus ISES Noseband Taper Gauge setting, with and without the DTG inserted, as well as a plot of the tension indicated on the DTG, are shown in Figure 16. The data for each arrangement fit a straight line over the ISES Noseband Taper Gauge range tested. Clearly, introducing the DTG probe changes the tension in the noseband, as does the ISES Noseband Taper Gauge (without the DTG). Based on the plots shown, the introduction of the DTG under the noseband is roughly equivalent to 1.7 fingers on the ISES Gauge, i.e., the DTG profile produces much the same tension as 1.7 fingers, as judged using the ISES Taper Gauge. The gradients of the linear fits to the data provide rates of change in tension per finger change on the ISES Gauge. These increase from 43 N/finger without the DTG inserted to 69 N/finger with it in situ. When the DTG is inserted, the DTG-indicated tension increases at the highest rate (84 N/finger). However, as Figure 16 illustrates, there is good correspondence between the DTG-indicated tension and the actual tension in the noseband over the range tested.

Noseband tension, as measured by the strain gauge, ranged from 3 N at 2 fingers to 36 N at 1.0 fingers’ space. Equivalent measures, recorded by the strain gauge while the Digital Tightness Gauge was under the noseband at the frontal nasal plane, ranged from 13 N at 2.0 fingers to 66 N at 1.0 fingers’ space. Noseband tightness, as measured by the Digital Tightness Gauge, ranged from 8 N at 2.0 fingers to 71 N at 1.0 fingers’ space.

## 4. Discussion

The purpose of the current study was to identify pressures, tensions, and data collection considerations across a range of noseband tightness settings. The first goal was to measure sub-noseband pressures at the frontal nasal plane, the left nasal bone, and at the second premolar tooth across the range of noseband tightness levels commonly used prior to the introduction of the FEI Measuring Device for Control of Noseband Tightness. The results of Test 1 revealed that pressure recorded at the frontal nasal plane, the location at which noseband tightness has traditionally been assessed, increased rapidly after the noseband was fastened tighter than Hole 10. This noseband tightness setting corresponds approximately with 1.4 fingers, as measured by the ISES Noseband Taper Gauge. An exponential increase in pressure occurs from that point (Hole 10) onward, as the noseband is tightened progressively to the tightest fitting, reaching 185 kPa. The initial slow increase in pressure up to that tipping point may arise as a result of shunting, i.e., preferential loading onto the prominent nasal bones on either side, until deformation of tissues effectively reduces their prominence such that the noseband starts to load the sensor at the midline position.

In a study of pressures beneath four different noseband types (cavesson, Swedish, flash, and drop), MacKechnie-Guire et al. (2024) also recorded steady increases (at the trot) in pressures when the noseband was tightened from two fingers of tightness to zero fingers of tightness [18]. The highest pressures recorded at the zero fingers’ tightness level in that study were much lower (12.1 kPa median maximal pressure under the Swedish noseband) than those found in the current study (383.3 kPa). Two factors may help to explain this disparity in values. In that study, where an array of 64 pressure sensors was used in a pressure mat (covering an area of 160 × 40 mm square extending to the side of the face, well beyond the bony prominences of left and right nasal bones), pressures were measured at 64 different points along this facial area spanning across both nasal bones, the concave area between the nasal bones and the lateral aspect of the face beyond the nasal bones. Pressures at the central midline and lateral to the nasal bones are lower due to the topography of the face at those locations (concave or flat at some points). Values from all sensors were summed, but the pressures reported do not isolate specifically the values at the location of interest in the current study, i.e., at the nasal bone or the mandibular rami. As has been identified previously [1,25], the lateral edges of the nasal bones (with their characteristic curvature) are the two points on the dorsal aspect of the face most likely to be subjected to the highest levels of pressure. The current study aimed to quantify those pressures.

Some identification of the patterns of pressure distribution exerted across the front of the face has been carried out [25,35]. While summing and averaging of values measured across the dorsal aspect of the face are of value and interest for comparative purposes (comparing different nosebands, pressures exerted during different activities), they do not identify the actual pressures at specific points of interest, as the data from areas of high and low radii of curvature are pooled. It would be interesting to investigate whether the data collected by sensors located at the left and right nasal bones by McKechnie-Guire et al. [18] would present similar pressure levels to those found in the current study.

In addition, in that study [18], the zero fingers’ tightness level used was defined as “the inability to admit the taper gauge beneath the noseband and no compression of the soft tissues”. To facilitate this, holes were punched at 0.5 cm intervals in the nosebands used in that study to allow for greater precision regarding different tightness levels. The inter-hole distance in the noseband used in the current study meant that the only option when going tighter than 0.5 fingers was to tighten the noseband by 1.5 cm, and this may have led to there being a greater difference in tension (and therefore pressure) between 0.5 fingers and zero fingers in the current study compared with that study. It is likely that this will have resulted in a difference between the definition of “zero fingers” in both studies (with “zero fingers” in the current study being tighter).

Data from the current study show exponential increases in sub-noseband pressures at settings tighter than 1.4 fingers’ space under the noseband, and this may also help to explain the substantially higher pressures recorded at the tightest noseband setting, as any increase in noseband tightness will have resulted in an exponential rather than linear increase in pressures.

In the current study, the substantial difference between pressures recorded at the midline frontal nasal plane location and the left nasal bone reflects the topography and profile of those locations, with a slight concavity frequently being present between left and right nasal bones that allows for some hammocking at this point. However, it is possible that, as the noseband is tightened, once the soft tissues have been fully compressed, the nasal bones themselves (and indeed other structures, such as the mandible) deform under the noseband’s applied load. This should result in an increased load to the midline pressure sensor, which is itself conformable but does have a finite height and so will, to a certain extent, fill the hollow (negative curvature region) between the nasal bones. The large pressure increases per hole seen for the midline beyond the hammocking region and the nasal bone would support this view.

The higher pressure found at the left nasal bone (which has a high radius of curvature) in comparison to the pressure at the frontal nasal plane is supported by the law of Laplace [28], where the same tensional force in the noseband is producing different levels of force against the underlying tissues, depending on the radius of curvature at that location (nasal bone vs. frontal nasal plane). The clear relationship shown between noseband tension and sub-noseband pressure, as predicted by the Law of Laplace, should allow regulatory authorities/riders to estimate sub-noseband pressures by measuring noseband tension, whether manually or using a specifically designed instrument.

The increase in pressures recorded at the left nasal bone and the second premolar tooth location appears to show a strong correlation; the rate of increase in pressures differs greatly, with an exponential increase in pressure recorded at the left nasal bone, and there is nearly a two orders of magnitude difference in pressure. Maximum pressures at the location of the second premolar tooth were relatively low (71.3 kPa), even at maximal noseband tightness. These relatively low pressures may have been due to lateral movement of the sensor during tightening of the noseband (and consequent shifting of tissues) off the surface of the second premolar tooth. A live horse would be expected to exceed the current pressures at this site as it chews spontaneously during ridden work and, therefore, moves its dental arcade laterally against buccal mucosa that is immobilised by the internal face of the noseband. To confirm, the current cadaver data represent the best-case scenario.

The highest pressure recorded at the left nasal bone, of 403 kPa, significantly exceeds pressure levels known to cause tissue damage and pain in both humans and animals [15,16,35]. MacKechnie-Guire et al. (2024) found that pressures at the mandibular rami are significantly higher than at the nasal bones, in many cases 3–4 times higher, raising alarm about the previously unmeasured pressures at this location and the need for regulation [18]. Due to limitations of the technology used in the current study, pressures at the mandibular rami were not measured.

Unlike pressures recorded as transient and pulsatile in other studies [1,25], pressures exerted at the nasal bones and mandibular rami are likely to be sustained at that location if nosebands are tight. Lateral excursions of the mandible would tend to produce a rotational force in the noseband relative to the horse’s nose. Such rotational forces will introduce shearing effects in tissue contacted by the noseband.

The results of Test 2 revealed that pressures for the left nasal bone again increased exponentially, after approximately 1.5 fingers (ISES Noseband Taper Gauge) of noseband tightness, reaching 383.30 kPa at the tightest noseband fitting. The exponential rise in pressure at approximately 1.4 fingers of noseband tightness demonstrates that, as compliance of the tissue is saturated and the noseband is effectively tightening against bone, pressure levels rise steeply. Occasional reductions in pressure can be seen (Table 3) at both the frontal nasal plane and left nasal bone at increasing levels of noseband tightness. These are most likely due to lateral movement of skin as the noseband was being tightened, resulting in movement of the sensor off the left nasal bone and possibly puckering skin at the frontal nasal plane in such a way as to angle the sensor and reduce the pressure applied to the pressure-measuring surface.

The second premolar tooth pressure data are again relatively low (185 kPa), reflecting the prospect of either cushioning of the sensor by the cheek tissue or movement of the sensor off the point of interest during tightening of the noseband. It should also be considered that the buccal mucosa is more easily damaged than skin and that stretched tissue will be thinner and, therefore, will be less resistant to pressure than unstretched tissue.

The findings of substantial increases in pressure and tension for each 0.5 finger increase in noseband tightness (Figure 13, Figure 14 and Figure 15) reflect other research findings in a study that involved eight live horses [18]. In that study, four types of nosebands were compared across five tightness levels, including post hoc tests for noseband type and noseband tightness. It failed to find a significant difference between 2.0 and 1.5 fingers, possibly due to the pooling of data from a variety of noseband designs. A potentially unforeseen consequence of that study is that the FEI has adopted a gauge with only 1.5 fingers’ space [27].

While the introduction of the Digital Tightness Gauge used in Test 3 artificially increased noseband tension, as measured by the strain gauge incorporated into the noseband, the impact on noseband tightness was uniform and so could be easily compensated/corrected. The output, measured in N, correlated with increasing noseband tension and may provide an objective measure of noseband tightness. Alternatively, for very tight nosebands, a Digital Tightness Gauge probe finger with a lower profile corresponding to half a finger could be used, but clearly it would be better if such tight nosebands did not occur.

It has been suggested that the use of very tight nosebands is comparable to the use of tourniquets on humans and animals [26]. Sustained pressure on living tissue is a concern in the use of tourniquets, where the duration of application and the level of pressure influence the likelihood and extent of tissue damage [15]. During surgery, tourniquets are routinely used to restrict blood flow to extremities [36,37], leading to recommendations regarding the optimal tensions and durations of use to optimise outcomes [16]. Adverse effects associated with the use of such tourniquets include nerve and soft tissue damage and pain, both during and following tourniquet use [15,36,37,38,39]. Neuron damage, muscle disabilities, and pain have been associated with sustained tourniquet pressures (6.6–46.7 kPa for 2 h), but briefer tourniquet application may also result in tissue damage [40,41]. While patients undergoing surgery are usually under general anaesthesia, an investigation into tourniquet-induced pain, subjecting 11 conscious healthy adult volunteers to tourniquet pressures of 39 and 53 kPa at the thigh [42], resulted in half of the interventions being terminated due to intolerable pain at the site of the tourniquet. Levy et al. recommend the lowest possible tourniquet pressure to maintain arterial closure in upper limb surgery, typically 13 kPa above systolic blood pressure [43]. Sarfani et al. support this recommendation, recommending an upper limit of 33 kPa during wrist surgery [44]. The use of tourniquets is generally a one-off event, unlike the use of nosebands, applied throughout daily or regular exercise and training, often for prolonged periods of time. Nosebands are typically tightened for the duration of exercise, which may last between 30 and 60 min and is likely to exceed the duration of tourniquets tightened to 32 kPa; for 20 min, they are used to elicit pain in horses experimentally [45].

While several in vivo studies measuring sub-noseband pressure levels on horses report cyclical changes in pressures recorded [18,25], if the lowest levels of pressure (i.e., the troughs) are sufficiently high to impair blood supply, via the vasa nervorum and vasa vasorum, small blood vessels that supply nutrients and oxygen to blood vessels and nerves of the underlying tissues and structures, and also the networks of capillaries supplying the periosteum, a tourniquet-type effect is possible, as this level of pressure will be sustained throughout exercise. The impact of recurring pressure peaks of the magnitudes reported in several studies [18,25] has not yet been studied. In noseband usage, while the contours of the face and location of major blood vessels prevent complete vascular restriction from occurring, localised pain due to direct pressure during usage at tightness levels resulting in this magnitude of pressure exertion at specific locations is highly likely. Although no data are currently available, there is concern that, indirectly, tight nosebands can compress the tongue and may lead to ischaemia, especially in the case of double bridles since two bits occupy more volume within the mouth than a single bit. Tight nosebands may mask pain, discomfort, and training methods that do not align with learning theory.

A small percentage of riding horses show deformation at the location of the noseband, accompanied, in some instances but not all, by loss of hair or changes in hair pigmentation. Arguably, such changes to the haircoat may just as easily arise from loose gear that rubs the integument. Retrospective analysis of radiographs of a group of horses investigated for a range of clinical abnormalities was inconclusive in its findings regarding nasal bone pathology [12]. Interestingly, a higher incidence of nasal bone abnormality was found in the warmblood horses within that study, possibly suggesting a link to particular riding activities (notably dressage) and the use of a noseband. In their study of 144 mature cavalry horses, [14] reported that 37.5% had one or more radiographic changes to the nasal bones, with radiological examination of these horses showing bone deposition (6.9%) and bone thinning (33.3%). Pérez-Manrique et al. (2020) reported that 13.8% of the same cavalry population had one or more radiographic changes to the mandible [14].

The periosteum has a dense network of sensory and sympathetic nerve fibres, with a greater density of innervation than any other part of the bone [29,42,43]. Nociceptors located in the periosteum are placed in the optimal location to detect pressure exerted on bone and transmit pain signals when stimulated through the exertion of pressure on bone [46]. Mechanical distortion of the periosteum is thought to be a major source of bone pain and is a contributory factor in pain following bone fracture [29].

The mean mechanical threshold for stimulation of nociceptors on a hind limb in cattle was shown by Ley et al. [47] to be 6.9 N. Forces required to stimulate nociceptors in sheep were 4.9 N [48]. The tightest noseband setting in the current study recorded noseband tension of 56 N (Test 2), which greatly exceeds those force levels. Tong et al. have shown that the epidermal innervation of equine and human skin from the gluteal region is remarkably similar, so the precautionary principle should certainly apply [49]. It is accepted that skin sensitivity and underlying tissue thickness affect horses’ responses to pressure over various anatomical landmarks. Similarly, the density of nociceptors varies across different anatomical regions. In horses without pre-existing painful pathology, mechanical nociceptive threshold values of 12 N/cm^2^ have been reported for the ventral abdominal wall [50].

The use of aversive stimuli in equitation is increasingly contentious. The horse reacts to pressure cues because the pressure is aversive. However, visible expression of pain is not easily detected in the horse [51]. Fortunately, progress is being made in the analysis of facial expression and changes in muscle tension at different locations of the face in response to procedures or situations expected to be painful or aversive in some other way [51]. The application of the Horse Grimace Scale or similar approaches to pain assessment, including deep learning from video inputs [45,52], may allow for the measurement of the aversiveness of the use of tight nosebands on horses in the future.

### Limitations

The authors acknowledge several limitations in the current study. First, we used no bit(s), so the pressures reported here should be considered the minimum for horse comfort when reins are not acting on the bit. The additional volume of bit(s) will occupy space in the buccal cavity and increase intra-oral pressures that are transmitted to the noseband when the horse opens, or attempts to open, the mouth. Second, in the absence of approval to deny any space under the noseband in a live horse, the current study was required to use a cadaver. The pressure from live tissues and the tongue’s inherent tendency to push against foreign bodies in the mouth would increase the tension through the noseband. So, again, we posit that the pressures reported here should be considered the minimum. Granting horses less than two fingers’ space is expected to compromise welfare as the intra-oral tissues, notably the tongue, are compressed when the horses cannot open their mouths. To apply learning theory correctly, if one chooses to use a noseband, there should be no pressure under the noseband unless the horse attempts to avoid bit pressure applied by the rider by opening its mouth rather than slowing. Third, the authors acknowledge that, when viewed laterally, the nasal profile of domestic horses varies [47]. So, on a pro rata basis, more space would be needed for comfort in horses with larger heads and more convex profiles than the head of the horse in the current study. Fourth, being primarily designed to identify two fingers’ space, as is traditional, with a secondary ridge to identify one finger’s space, the ISES Noseband Taper Gauge should be regarded as only indicative when assessing 0.5 and 1.5 fingers’ space. Fifth, it is acknowledged that the ISES Noseband Taper Gauge could be inserted with greater or lesser amounts of force to render different outcomes. Every attempt was made to minimise variation in the amount of force used, but the estimates made are unavoidably approximate rather than precise measurements. Sixth, this was a study using a single cadaver head with a single type of noseband/bridle, using tissue that had undergone a freeze–thaw cycle and was subject to none of the changes associated with equine locomotion, some of which create a cyclical pattern of pressures. In addition, the morphology of horse heads varies greatly, and the current use of a single specimen limits the extent to which we can draw conclusions for all horses.

Finally, we note that other bridles will have gaps of different lengths between holes on their nosebands. For that reason, and to overcome the indicative nature of the fractional spacings (see above), we studied the ISES Noseband Taper Gauge spacings that were closest to the existing holes in the current bridle.

## 5. Conclusions

The origin and basis for the traditional two-finger guideline is unknown, but the rapid escalation in pressure measured at the nasal bone revealed in this study, from approximately 1.4 fingers noseband tightness level, emphasises the merit of the two-finger guideline in protecting the nasal bones from pressures that may compromise the welfare of the ridden horse. Given that pressures are expected to rise from those reported here when horses wear bits, locomote, and when the reins are under tension, we conclude that, even though this study was conducted on a single cadaver head with a single type of noseband, the traditional provision of two fingers of space should be retained.

## Figures and Tables

**Table 1:** Approximate correspondence between noseband Holes 6–14 and the ISES Noseband Taper Gauge estimate. The ISES Taper Gauge showed 2.0 fingers’ tightness at noseband hole No. 7, with reducing estimates of space under the noseband at each consecutive hole number, with the noseband too tight to allow for insertion of the ISES Taper Gauge at hole No. 14 (zero fingers).

ISES Noseband Taper Gauge	Noseband Hole Number
Zero fingers	14
0.5 fingers	13
1.0 fingers	12
1.2 fingers	11
1.4 fingers	10
1.5 fingers	9
1.7 fingers	8
2.0 fingers	7
Loose	6

**Table 2:** Test 1 results: Pressure (kPa) exerted by the noseband at the frontal nasal plane, at the left nasal bone, and at the second premolar tooth (with the sensor facing the buccal mucosa) at 8 noseband tightness fittings.

Noseband Hole Number	Frontal Nasal Plane (kPa)	Left Nasal Bone (kPa)	Premolar 2 (kPa)
6	−0.01	−0.01	0.071
7	−0.01	20.27	0.16
8	−0.00	79.07	0.64
9	0.55	167.67	2.26
10	0.61	192.30	3.43
11	23.29	263.45	5.34
12	96.61	291.37	5.44
13	185.21	403.19	7.13

**Table 3:** Test 2 results: Pressure (kPa) exerted by the noseband at the frontal nasal plane, at the left nasal bone, and at the second premolar tooth (sensor facing tooth surface). Also, tension is measured by the inbuilt noseband strain gauge (N) at 9 tightness fittings, from noseband hole No. 6 to hole No. 14.

Noseband Hole Number	ISES Noseband Taper Gauge	Frontal Nasal Plane (kPa)	L Nasal Bone (kPa)	Premolar 2 (kPa)	Noseband Tension (N)
6	Loose	1.73	5.33	0	1
7	2 fingers	4.80	2.93	0.4	2
8	1.5 fingers	102.52	1.07	0.93	6
9	1.3 fingers	138.65	7.33	2.4	10
10	1 fingers	91.86	43.20	3.20	14
11	0.7 fingers	120.65	78.92	5.47	22
12	0.5 fingers	83.06	236.25	6.80	27
13	0.2 fingers	123.59	382.37	11.07	37
14	0 fingers	178.91	383.30	18.53	56

## Data Availability

Data that support the current conclusions are available upon request.
